# Supplementary material for: Selection of HIV-1 for resistance to fifth-generation protease inhibitors reveals two independent pathways to high-level resistance
Source: eLife. 2023 Mar 15;12:e80328. doi: 10.7554/eLife.80328 (PMC10065791; doi:10.7554/eLife.80328)
Supplement: Transparent reporting form [file elife-80328-transrepform1.pdf]

eLIFE
1st Floor
24 Hills Road
Cambridge CB2 1JP, UK
P 01223 855340
W elifesciences.org
T @elife

eLife's transparent reporting form

We encourage authors to provide detailed information *within their submission* to facilitate the interpretation and replication of experiments. Authors can upload supporting documentation to indicate the use of appropriate reporting guidelines for health-related research (see EQUATOR Network), life science research (see the BioSharing Information Resource), or the ARRIVE guidelines for reporting work involving animal research. Where applicable, authors should refer to any relevant reporting standards documents in this form.

If you have any questions, please consult our Journal Policies and/or contact us: editorial@elifesciences.org.

Sample-size estimation

- You should state whether an appropriate sample size was computed when the study was being designed
- You should state the statistical method of sample size computation and any required assumptions
- If no explicit power analysis was used, you should describe how you decided what sample (replicate) size (number) to use

Please outline where this information can be found within the submission (e.g., sections or figure legends), or explain why this information doesn't apply to your submission:

Sample sizes were decided from experience of the techniques performed and practical considerations. n Numbers and statistical tests are given in figure legends.
Sample sizes are indicated in the figure legends where appropriate
1) Data averaged for individual passages, with each passage being a single n, but generates large amounts of reads that return an average of 10,000 sequences to analyze. These ~10,000 sequences at each timepoint were used to analyze abundance and entropy data down to 0.1%.
2) R1 and R2 groups used to analyze drug sequencing and selection data were selected and identified based on drug structure, so some groups have an n of 10 through both experiments, and some smaller subgroups analyzed only contain 4 total measurements.
3) For figure 4, the sample numbers are given for each group, but 3 cultures were excluded from the graph because they had both I50V and I84V fixed in the culture. (V2mut, V2wt, V5wt)
4) For figure 6, the sample numbers are 50 for A-B, but 40 for C-E due to 84V and 50V being fixed in the same culture for those excluded.

Replicates

- You should report how often each experiment was performed
- You should include a definition of biological versus technical replication
- The data obtained should be provided and sufficient information should be provided to indicate the number of independent biological and/or technical replicates
- If you encountered any outliers, you should describe how these were handled
- Criteria for exclusion/inclusion of data should be clearly stated
- High-throughput sequence data should be uploaded before submission, with a private link for reviewers provided (these are available from both GEO and ArrayExpress)

Please outline where this information can be found within the submission (e.g., sections or figure legends), or explain why this information doesn't apply to your submission:

Replicate numbers were decided from experience of the techniques performed and practical considerations The number of all biological replicates will be given in the figure legends, or are otherwise a single measurement. Replicates used to obtain EC50 values were of 3 independent infections measured with 3 technical replicates in the luminometer. Around 10% of the EC50 inhibition curves had one point where the value was >125% infectivity of the first point. Anomalous values were excluded from curve fitting. 4 curves did not record data.

Statistical reporting

- Statistical analysis methods should be described and justified
- Raw data should be presented in figures whenever informative to do so (typically when N per group is less than 10)
- For each experiment, you should identify the statistical tests used, exact values of N, definitions of center, methods of multiple test correction, and dispersion and precision measures (e.g., mean, median, SD, SEM, confidence intervals; and, for the major substantive results, a measure of effect size (e.g., Pearson's r, Cohen's d)
- Report exact p-values wherever possible alongside the summary statistics and 95% confidence intervals. These should be reported for all key questions and not only when the p-value is less than 0.05.

Please outline where this information can be found within the submission (e.g., sections or figure legends), or explain why this information doesn't apply to your submission:

General information on data analysis given at end of Methods section. Every figure legend has statistical information concerning the exact values of N, data representation, and details of tests used. P-values are represented by a significance values on asterisks. Nonsignificant p-values are shown with no bracket above them.

(For large datasets, or papers with a very large number of statistical tests, you may upload a single table file with tests, Ns, etc., with reference to sections in the manuscript.)

Group allocation

- Indicate how samples were allocated into experimental groups (in the case of clinical studies, please specify allocation to treatment method); if randomization was used, please also state if restricted randomization was applied
- Indicate if masking was used during group allocation, data collection and/or data analysis

Please outline where this information can be found within the submission (e.g., sections or figure legends), or explain why this information doesn't apply to your submission:

NA

Additional data files ("source data")

- We encourage you to upload relevant additional data files, such as numerical data that are represented as a graph in a figure, or as a summary table
- Where provided, these should be in the most useful format, and they can be uploaded as "Source data" files linked to a main figure or table
- Include model definition files including the full list of parameters used
- Include code used for data analysis (e.g., R, MatLab)
- Avoid stating that data files are "available upon request"

Please indicate the figures or tables for which source data files have been provided:

The sequencing data (Figure 2, 3, and 4) is available at NIH Sequencing Read Archive (SRA) under BioProject ID PRJNA853351.

All source data files for enzymatic Ki and Km (Table 1, Figure 2 and 5) have been uploaded to the Carolina Digital Repository: Swanstrom, Ron, and Ean Spielvogel. Km and Ki Dataset for Selection of Hiv-1 for Resistance to Fifth Generation Protease Inhibitors Reveals Two Independent Pathways to High-level Resistance. 2022.

All source data files for EC50 inhibition curves (Table 1, Figure 2 and 6) have been uploaded to the Carolina Digital Repository: Swanstrom, Ron, and Ean Spielvogel. Ec50 Dataset for Selection of Hiv-1 for Resistance to Fifth Generation Protease Inhibitors Reveals Two Independent Pathways to High-level Resistance. 2022.
